# The Opportunities and Challenges of Digital Anatomy for Medical Sciences: Narrative Review

**DOI:** 10.2196/34687

**Published:** 2022-05-20

**Authors:** Nilmini Wickramasinghe, Bruce R Thompson, Junhua Xiao

**Affiliations:** 1 School of Health Sciences Swinburne University of Technology Victoria Australia; 2 Epworth Healthcare Melbourne Australia; 3 Alfred Health Melbourne Australia; 4 School of Health Sciences University of Melbourne Parkville Australia; 5 School of Allied Health La Trobe University Bundoora Australia

**Keywords:** digital anatomy, digital health, virtual reality, augmented reality, medical education

## Abstract

**Background:**

Anatomy has been the cornerstone of medical education for centuries. However, given the advances in the Internet of Things, this landscape has been augmented in the past decade, shifting toward a greater focus on adopting digital technologies. Digital anatomy is emerging as a new discipline that represents an opportunity to embrace advances in digital health technologies and apply them to the domain of modern medical sciences. Notably, the use of augmented or mixed and virtual reality as well as mobile and platforms and 3D printing in modern anatomy has dramatically increased in the last 5 years.

**Objective:**

This review aims to outline the emerging area of digital anatomy and summarize opportunities and challenges for incorporating digital anatomy in medical science education and practices.

**Methods:**

Literature searches were performed using the PubMed, Embase, and MEDLINE bibliographic databases for research articles published between January 2005 and June 2021 (inclusive). Out of the 4650 articles, 651 (14%) were advanced to full-text screening and 77 (1.7%) were eligible for inclusion in the narrative review. We performed a Strength, Weakness, Opportunity, and Threat (SWOT) analysis to evaluate the role that digital anatomy plays in both the learning and teaching of medicine and health sciences as well as its practice.

**Results:**

Digital anatomy has not only revolutionized undergraduate anatomy education via 3D reconstruction of the human body but is shifting the paradigm of pre- and vocational training for medical professionals via digital simulation, advancing health care. Importantly, it was noted that digital anatomy not only benefits in situ real time clinical practice but also has many advantages for learning and teaching clinicians at multiple levels. Using the SWOT analysis, we described strengths and opportunities that together serve to underscore the benefits of embracing digital anatomy, in particular the areas for collaboration and medical advances. The SWOT analysis also identified a few weaknesses associated with digital anatomy, which are primarily related to the fact that the current reach and range of applications for digital anatomy are very limited owing to its nascent nature. Furthermore, threats are limited to technical aspects such as hardware and software issues.

**Conclusions:**

This review highlights the advances in digital health and Health 4.0 in key areas of digital anatomy analytics. The continuous evolution of digital technologies will increase their ability to reinforce anatomy knowledge and advance clinical practice. However, digital anatomy education should not be viewed as a simple technical conversion and needs an explicit pedagogical framework. This review will be a valuable asset for educators and researchers to incorporate digital anatomy into the learning and teaching of medical sciences and their practice.

## Introduction

### Background

For over 10 years, the relatively nascent domain of digital health [1,2] and the application and embracement of the tools, techniques, and technologies of Industry 4.0 into health care delivery have advanced and matured [3,4]. Much of this advancement has been because of the breakthroughs in the technologies making up the Internet of Things, including mobile and platforms, virtual reality (VR), mixed reality (MR) and augmented reality (AR), 3D printing, analytics, and sensors [3]. Taken together, these technologies are serving to affect the digital transformation of health care delivery [4]; that is, making the interaction with technology, be it mobile solutions, reliance on analytics, sensors, AR, VR, or MR, an integral part of patient and clinician activity in either the receiving or delivery of care. This is being done to support a health care value proposition of better access to care and a higher quality of care and to ensure that a high value of care ensues [5].

In this context, it is natural to see a similar transformation in various aspects of the medical field [6,7]. To date, notable advances in medicine which uses these technologies include orthopedics where 3D printing is now being used to replace or repair body parts such as a broken jaw; robotics is used to facilitate minimal invasive surgery; and analytics visual, imaging, or text in particular is being used extensively in cancer care [8]. One area that has been slow to embrace technological advances has been anatomy, a field that is of critical importance to medicine and the delivery of health care. Today, in most medical schools, anatomy is taught in the traditional fashion with cadavers [9-11], and only a few leading medical schools are venturing into the domain of digital anatomy, where the technologies of the Internet of Things, especially VR, AR, and MR, are used to recreate human structures to support the study of the human body.

Specifically, digital anatomy, or what is often defined as computer-based 3D modeling of the human body [12], is an area of growing importance and significance. Digital anatomy is advancing and becoming more sophisticated given the progress and sophistication in computer modeling, VR, MR and AR. Moreover, the benefits of digital anatomy appear to be far-reaching and provide assistance to students, clinicians, patients, and other stakeholders [12]. In addition, digital anatomy provides a cost-effective approach to realizing high-quality outcomes [13]. This study contends that digital anatomy represents an opportunity to embrace advances in digital health technologies and apply them to the domain of anatomy to enhance and modernize this core area of medical science. This review aims to outline the emerging area of digital anatomy in both teaching and research and importantly summarize opportunities and challenges for incorporating digital anatomy in medical science education and practices, which is particularly pertinent to future medical and health science education. The following then serves to answer the following research questions: “What are the barriers and facilitators for the implementation of the digital technology in anatomy education and research,” “How can we embrace digital health technologies to advance the domain of anatomy,” and “What are the strengths, weaknesses, opportunities, and threats in which may arise when digital health technologies are incorporated to advance the domain of anatomy?”

### Background of Digital Anatomy

Anatomy has over 2000 years of history [14-16] with the first documented scientific description of human structures by Hippocrates of Cos (V-IV centuries BC) [17]. Although *ancient*, this scientific discipline is *born* to be highly adaptive and has undergone checkered changes from its beginning in ancient Greece, Renaissance, to the 19th and now the 21st century [16,18-20]. Modern anatomy teaching is now embracing new innovative modalities. Although human cadavers are viewed as the gold standard for best practices in anatomical education, there is a clear shift from traditional, cadaver-based anatomy teaching toward digital tools–based or mixed (digital tools plus cadavers) curriculum [12,21]. This shift is inevitable, not only driven by an increase in student numbers and financial and ethical constraints on cadaver use but also by the rapid development of medical technology. Digital anatomy is emerging as a new discipline [12,22], representing an intersection of converging disciplines, including medical imaging, 3D reconstruction and printing, AR, and artificial intelligence and robotics.

### Digital Anatomy Education Is Fast Developing

An array of digital anatomy tools is currently available with supplementary features [23] and provides curriculum developers with more opportunities to achieve the desired learning outcomes. VR, AR, MR, 3D printing, and tablet-based programs are digital approaches commonly used worldwide for teaching gross and regional anatomy [12,23] either on-site or on the web (web- or cloud-based). When combined to teach the same structures, VR, AR, and tablet anatomy apps have been found to increase learner immersion and engagement [24], and learners develop a deeper understanding of surface anatomy and internal structures relative to their surroundings [25], the latter of which is a desired anatomy learning outcome.

One genuine concern, however, is whether digital anatomy is adequately sufficient for medical education. It is important to appreciate that digital anatomy tools have become more sophisticated, and their fidelity has improved compared with what they were 10 or 5 years ago [12]. Recent digital anatomy resources provide comparative learning outcomes such as understanding of disease and pathology to students, including medical students, compared with cadaver-based education [26-29]. Over 75% of anatomy pedagogy research (126 out of 164 studies) conducted between 2007 and 2017 found that digital technologies enhance anatomical education across multiple disciplines, including medicine, surgery, dentistry, and allied health professions [30]. Virtual or digital dissection tables have been effectively implemented in medical education, providing an alternative experience for understanding human body complexity and layers of internal structures. Junior medical students perceive the use of virtual dissection as a valuable tool for learning anatomy and radiology [27]. Evaluation of the effectiveness of anatomical education using digital technologies, including AR, VR, and MR, supports several pedagogy measurements, including student experience and satisfaction [23,24,28,30,31], learning performance and outcome [25-27,29,31,32], problem-solving skills and clinical reasoning [32,33], and postintervention knowledge and skills outcomes [34-36], with or without comparison with traditional teaching. Overall, these studies support the premise of applying digital anatomy as a means of curriculum development for more surgical-oriented training. The development of digital anatomy cannot replace cadaver-based anatomy teaching but offers unique and sustainable learning experiences and outcomes. Hence, the question of whether digital technology can be used in anatomical education has passed and curriculum developers have now reached a new phase of how to effectively apply digital modalities to learner-centered education that best suit course design and learning outcomes.

Today, digital approaches are combined with blended teaching modes in both face-to-face and remote learning and case- and group-based studies, fostering interdisciplinary collaboration. However, effective digital anatomy education requires curriculum redesign. Digital anatomy education should not be viewed as a simple technical conversion from cadaveric to digital approaches. When designing a digital anatomy curriculum, regardless of the discipline, content delivery needs to be tightly linked with the chosen digital tools. Careful alignment between learning tasks and performance measures using digital tools is required [37]. Different digital anatomy tools come with supplementary features; hence, their selection needs to best meet the graduates’ attributes. Moreover, anatomy education, either virtual or classical, requires trained personnel and time. However, the decline in trained anatomy instructors is an ongoing challenge in this discipline [14]. When moving into the digital phase, the competence and attitude of the staff toward using digital tools in teaching and research is another key factor in the success of digital anatomy course delivery and development.

### Digital Anatomy Research Is Moving Into a New Dimension

Anatomy is far more than *landmarks*. Many medical advances have been made in anatomical research. Specialized medical imaging techniques such as computed tomography scans and magnetic resonance imaging have revolutionized health care service quality through better visualization of patients’ anatomy, including pathology. Together with physicians, surgeons, radiologists, and computational scientists, anatomists can now adopt the latest digital technologies to promulgate a questioning scientific spirit. The human body is a typical example of organism diversity and variation, but 2D slices are no longer sufficient to provide a full picture that can guide clinical management and regimes. In this context, digital anatomy research driven by clinical problems has established a new research dimension in this discipline. 3D-printed anatomy models not only function as a training tool for both health professionals and patients [38,39] but also advance the development of tissue engineering [40-42] and patient-specific medical devices [43,44], which have lifesaving potential for complex cases. Moreover, the digital processing of anatomical data provides a precise representation of the patient’s anatomy. Indeed, the digitization of patient-specific anatomy has gained momentum in recent years [45-47] and has moved beyond 3D printing [46,48]. It is envisaged that patient-specific digital anatomy could be a catalyst for changing and bridging the quality of personalized care. AR merges virtual patient-specific anatomy into a real surgical view. By adopting 3D modeling and preoperative virtual planning, anatomists can generate 3D reconstructed organs for individual patients before surgery [45,46] and enable statistical modeling that can accurately predict posttraumatic or postsurgical conditions [47]. Therefore, patient-specific anatomy will allow for more accurate risk evaluation of invasive surgery [47,49] and provide advanced personalized patient care. In this context, digital anatomy will revolutionize our understanding of anatomical variations and effectively apply them in clinical settings. From a training perspective, this new field of digital anatomy research may also enable the rehearsal of virtual surgeries, such as virtual transplantation. In the future, patient-specific digital anatomy research could be successfully integrated with big data analytics and deep learning [8]. In so doing, the outcomes of digital anatomy research will be transformational, not only applying to personalized surgeries for many organs and digital health but also providing unprecedented insights into health care advancement.

### Digital Anatomy in Clinical Practice: A Shifting Paradigm for Future Health Care Education

The rapid development of digital anatomy will not only revolutionize undergraduate anatomy education but also shift the paradigm of pre- and vocational training for health care professionals. New clinical skills that require digital technology have evolved for advanced treatments [50]. However, restricted operative opportunities for medical and surgical trainees remain a challenging issue in clinical training around the world because of financial, resource, and other logistical constraints and the recent COVID-19 pandemic [51]. Furthermore, traditional cadaver-based clinical training does not address the need for growing health care advancements. Thus, efforts have been made to provide digital-based operative education outside the theater and cadaver laboratory, while reducing the variability in trainees’ operative experience and skills.

Digital-based clinical simulation has been delivered for new and advanced practices, with consistent positive effects on knowledge, skills, and professional behaviors. Digital simulations with improved fidelity such as haptic features are being applied at all levels of learners to a wide range of surgical training, including ear, nose, or throat [52,53]; orthopedic [54]; vascular [55]; ophthalmic [56-58], and neurosurgeries [59-63], involving endoscopy [61,64-68], laparoscopy [69], and robotics [8]. The AR or VR anatomy teaching of health professionals shows clear benefits of improving surgical confidence [70], performance [63], and postintervention knowledge and skills [34-36], particularly in complex conditions such as neurological and cardiovascular diseases [33]. These studies demonstrate that digital simulation for surgical anatomy training is feasible and provides depth perception of surgical procedures, which is particularly pertinent for training novice or inexperienced surgical residents [63,71]

Moreover, a virtual surgical simulator has been developed for cleft repair surgical education [72,73]. Virtual technology has been found to increase nursing students’ clinical skills without harming patients and help prepare nurses for new practices such as robotic surgery [74]. Together, digital-based clinical skill training has been established as a new training model for health professionals, improving postintervention knowledge and skill outcomes via a virtual and immersive environment when compared with traditional education [36].

Modern health care practitioners, from nurses to physicians and allied health professionals, are required to work effectively in teams and rely on each other’s expertise to provide holistic and optimal patient-centered management. Digital health has substantially improved the way health professionals handle clinical phases. Recent advances in the digital acquisition of patient-specific anatomy data can provide substantial information to the clinician; hence, complex surgeries and less invasive local therapies can be easily planned [45-47,49]. Thus, digital anatomy–based clinical training and practice will continue to address restrictions and reduce disparities in surgical training. This is expected to have a significant impact on future precision surgery and development of collaborative global curricula in surgical education. It is possible that digital anatomy laboratories may become part of the operation in future hospitals, and health professionals in different disciplines, including medical and surgical residents and fellows, could review regions pertinent to their specialties.

## Methods

**Figure 1:**
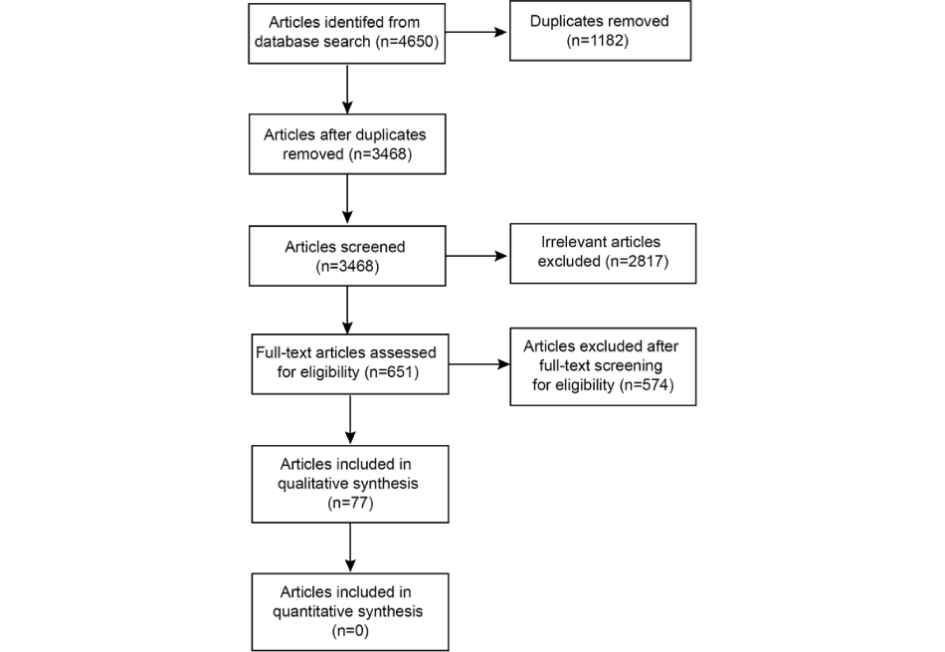
A summary of selected articles shown in the PRISMA (Preferred Reporting Items for Systematic Reviews and Meta-Analyses) flow diagram.

Literature searches were performed using the PubMed, Embase, and MEDLINE bibliographic databases for research articles published between January 2005 and June 2021 (inclusive; see Figure 1 for the search flow diagram). Combinations of the following search terms and subheadings were considered appropriate for this investigation: *anatomy, digital anatomy, virtual reality, augmented reality, mixed reality, apps, teaching, education, anatomy,* and *training.* The publications chosen were restricted to those written in English that described human anatomical education or training within the health and medical sciences. Articles published in the fields of nonhuman anatomy education and microscopic anatomy were excluded from the study. The primary aim of this study was to identify the barriers to and facilitators for the implementation of digital anatomy education and research. No ethical clearance was required for this study because all selected studies had previously received ethics approval from local institutional review boards. A Strength, Weakness, Opportunity, and Threat (SWOT) analysis (descriptive analysis) was performed to evaluate the roles that digital anatomy plays in both the learning and teaching of medicine and health sciences as well as its practice. A formal meta-analysis was not performed owing to the heterogeneity of the retrieved data.

## Results

### Overview

A move toward digital anatomy is inevitable. As with the development of any new discipline, there are both strengths and weaknesses associated with digital anatomy, accompanied by potential threats and opportunities, as shown by the SWOT analysis (Table 1).

**Table 1:** Matrix analysis of digital anatomy.

Strategy	Strength	Weakness	Threat	Opportunity
Cost	Cost-effective and low maintenance over the long term (personnel and resources) [13]	Considerable initial setup cost such as hardware devices	—^a^	Digital anatomy resources more accessible
Education setting	Flexible; time-efficient; low maintenance required for manual handling and occupational safety	Virtual; digital tools not always compatible with existing infrastructure or teaching settings in the cadaver laboratory	Hardware and software upgrade; the cost of changing; conservative thinkers	New technical development and software upgrade available; compatible with increase in student numbers and the demand of remote learning; new options for future clinical skill laboratory in the hospital
Learner experience	Combine surface and regional anatomy [23]; consistent learner satisfaction [23,24,28,30,31]; better visualizing deeper structures incorporating virtual dissection; integrating anatomy, physiology, and pathology; integrating gross and microscopic anatomy with medical imaging in one setting	Currently limited on showing anatomical variations; current virtual dissection has lack of tactile information; shortfall in learner-centered digital technologies in health care education [32]	Variable digital competencies of users (instructor and student) [30]; limited exposure to human body variability	Augmented reality and virtual reality resources more sophisticated [12] with supplementary features [23]; digital and haptic technologies are being integrated for surgical anatomy training [75,76]; embrace anatomy learning with new medical technology; enable discipline-specific learning [64,65]
Learning outcome	Enable streamed group-based study on the same anatomical structure (not possible on a single cadaver or model) [23]; cognitive skills and memory retention; postintervention knowledge and skills outcomes [36]; unique attributes to safe clinical practice [74]; improve clinical reasoning [32,33,77]	Currently lacked explicit pedagogical framework	Limited education opportunity for learners’ feelings about death; potential lack of traditional surgical skills training; impact of new digital anatomy curricula on future surgical competencies unclear	Allow vertical integration of surgical anatomy through advanced curricula; enable training for new and advanced practices [74,78]; advance patient-specific anatomy for personalized health care and training [46]; learner-centered health care education [32]
Collaboration and medical advances	Accessible for users’ self-revision; enable flexible and rapid curriculum change; address restrictions and reduce disparities in surgical training; improve informed patient consent and education for surgical planning [38,39]; enable and catalyze resource sharing and collaboration at all levels of training and practice	—	—	Enable sophisticated preoperative study [45-47,49]; trigger curriculum redesign; foster new collaborative graduate courses [79]; catalyze new specialties and medical advances; advance personalized patient health care [43,45-47]; integrating into digital health

^a^No data available.

### Cost and Class Setting

From an economic perspective, the implementation of digital anatomy has a clear and cost-effective impact. The use of digital techniques would dramatically reduce the ongoing cost spent on classes, books, mannequins, cadavers (procurement, preparation, and disposal), and ventilation systems compared with traditional anatomy teachings, estimated at US $390,000 every 5 years [13]. Moreover, the number of digital technology options continues to increase. Hence, the cost of such equipment is likely to decrease over time because of the rise in competition. Although there is considerable initial setup cost, particularly for hardware devices, the overall maintenance is low over the long term [13]. Moreover, digital anatomy laboratories require minimum manual handling and ventilation monitoring because of occupational fixative exposure, both of which are ongoing work-related safety hazards in cadaver laboratories. Thus, the class setting and turnover of the digital anatomy laboratory are flexible, time-efficient, and readily accessible for both learners and instructors. In addition, many digital anatomy resources allow web- or cloud-based access, thereby allowing the development and delivery of new courses for remote learning without significant administrative investment. In light of the lengthy global pandemic, digital anatomy represents unique advantages for class setting change, resource development, and new course development during both the COVID and post-COVID periods. The rapid development of digital technology together with patient-specific anatomy research will make virtual surgical planning and rehearsal room possible in the future [46]. Overall, digital anatomy can provide cost-effective education at a time when the demands on training and health care services continue to increase.

### Medical Education: Learning Experience and Outcome

Digital technology–based education has demonstrated consistent learner satisfaction, outperforming the traditional approaches [23,24,28,30,31]. However, representations of human body variations are limited to the current digital resource capacities. Thus, there is a need to expand the repertoire of digitized cadaveric resources, particularly for the appreciation of human body variations and diversity. New digital resources are being rapidly developed and have shown a good correlation between cadaveric materials and digital resources, such as for coronary artery distribution [80]. When combining AR with virtual dissection, digital anatomy has clear advantages for visualizing internal deeper structures and integrating anatomy, physiology, pathology, and medical imaging for the same structure at both gross and microscopic levels (Table 1). This ultimately enables a streamed group-based study of the same anatomical structure, which is not possible with a single cadaver or model [23]. When implemented for simulation classes, blended digital anatomy teaching enhances postintervention knowledge and skills [36].

Competent clinicians, particularly surgeons, need a deep understanding of anatomy for safe clinical procedures. In this context, digital anatomy has unique attributes for clinical training, such as patient safety, postintervention knowledge and skill outcomes, and real-life conditions without time limitations and patient discomfort [36,74]. However, there is a concern that medical students without exposure to cadaver-based dissection could be less competent in surgical skills and have limited opportunities for learning about death; hence, they are poorly prepared when entering clerkships and residency programs. The cadaver-based dissection class, limited by the number of bodies, means to preserve them, and associated logistics, is an ongoing and worldwide challenge in medical and surgical education. In this context, virtual or digital dissection provides an alternative and valuable learning experience for medical students [27] when classic dissection classes are not accessible. Moreover, the ongoing and rapid development of sophisticated digital tools with high fidelity, such as digital anatomy education incorporating haptic technologies [76], will significantly advance new clinical practices such as robotic surgery for health professionals.

That said, the impact of new digital anatomy curriculum reforms on the retention of future surgical competencies is currently unclear (Table 1). Therefore, future research is required to evaluate students’ perceptions and effectiveness of digital simulation in the satisfactory development of surgical skills. In addition, proficiency in surgical care is complex, as it not only involves knowledge of instrumentation and surgical procedures but also a comprehensive integration of anatomy and physiology for the organ being operated. In this context, digital anatomy resources provide sophisticated tools for preoperative study, particularly complex surgical planning [45-47,49] (Table 1). Although digital anatomy has unique attributes that can improve learning outcomes when compared with traditional learning methods [32], there is currently a shortfall in learner-centered implementation of digital technologies in health care education, where these technologies have the capacity to cause a paradigm shift (Table 1).

The digital competencies of users (both instructors and students) and their attitude toward technology is another key element that heavily influences curriculum effectiveness (Table 1). Researchers might be overconfident in the use of digital technologies [30], highlighting the importance of adequate technical training for instructors. VR and AR technologies display supplementary features and suit different purposes of anatomy teaching and research (Table 1). VR was found to be the most prevalent and influential digital technology, followed by web-based and computer-aided resources [30]. Although AR and VR are relatively mature technologies suitable for surface and regional applications, MR is still a developing technology and is not necessarily consumer-ready at this point of time [23]. However, research in the educational setting shows great promise in its potential to allow multiple users to visualize the same structure if the headsets communicate with each other [23].

## Discussion

Using SWOT analysis (Table 1), we described strengths and opportunities that together serve to underscore the benefits of embracing digital anatomy, particularly in the areas of medical education and advances. The SWOT analysis also identified a few weaknesses associated with digital anatomy, which are primarily related to the fact that the current reach and range of applications for digital anatomy are very limited because of its nascent nature. Furthermore, threats are limited to technical aspects such as hardware and software issues. This finding highlights the advances in digital health and Health 4.0 in key areas of digital anatomy analytics.

Digital health is still quite nascent; however, the benefits of the application of advances in technology and the development of technologies that make up the Internet of Things to health care are significant and difficult to quantify. Many aspects of digital health, such as telehealth, have recently become very important given the recent COVID pandemic but before this, although recognized as having many advantages, had yet not received universal appeal [4]. It is in this context that applying such advances to the area of anatomy, the domain that has for the most part remained quite traditional in not only the way it is taught to medical students but also in how clinicians typically refer to anatomy in clinical consultations or in the operating theater is considered. Specifically, the broadening of anatomy is recommended to include consideration of digital anatomy. From a technical perspective, digital anatomy is not a major challenge given that the technologies of AR, VR and MR are well developed and easily transferable to this context as are other techniques and technologies around analytics and even 3D printing. However, digital anatomy education should not be viewed as a simple technical conversion and needs an explicit pedagogical framework. It is more challenging to apply these technological advances to relatively traditional domains. This requires changes in the processes and mindsets of key stakeholders, policymakers, regulatory bodies, and advocate groups. We assert that it is necessary to embrace digital advances in anatomy and incorporate digital anatomy into various contexts such as education and clinical practice so that it will be possible to realize the benefits, strengths, and opportunities identified in Table 1 that digital anatomy affords. This will serve not only to advance digital health but also to provide a better educational experience and understanding of anatomy for clinicians, which in turn will translate into better patient outcomes.

The preceding section outlined the emerging area of digital anatomy. This was done by first highlighting the advances in digital health and Health 4.0 in key areas of analytics and AR, MR and VR as well as mobile and platforms and 3D printing. From this, the impact of analytics and AR, MR and VR in particular can be harnessed to enable the vision of digital anatomy to be realized. Importantly, it was noted that digital anatomy cannot only benefit in situ real time clinical practice but also has many advantages for learning and teaching clinicians at multiple levels.

To unpack this further, we presented a SWOT analysis of opportunities for incorporating digital anatomy. It is apparent from Table 1 that there are very few weaknesses with digital anatomy, most of which are related to the fact that the current reach and range of applications for digital anatomy are very limited because of its nascent nature. Furthermore, the threats are limited to technical aspects such as hardware and software issues; given that hardware and software costs not only continue to decrease over time but also witness significant improvements and advances, we are of the opinion that this threat, although minimal, will diminish further in the fullness of time. The continuous evolution of digital technologies will increase their ability to reinforce anatomy knowledge and advance clinical practice. Similarly, we are confident that digital literacy and proficiency of students and clinicians will increase over time. Moreover, we are confident that expectations from students and clinicians regarding digital anatomy will increase over time. What is particularly pleasing is that Table 1 clearly highlights many and multiple strengths and opportunities that taken together serve to underscore the benefits of embracing digital anatomy. Future research should evaluate the long-term effectiveness of digital anatomy and its impact on multiple domains, such as changes in learners’ practice, behavior, and skills. There are also some limitations associated with this study. Qualitative analysis was performed on the included studies; however, a meta-analysis was not performed because of the heterogeneity of the retrieved data.

We set out to create a case for digital anatomy. Table 1 lists its merits and benefits. However, that is in reality only the beginning. For digital anatomy to be fully embraced and realized, it is necessary for policy to be affected to encourage and support its adoption. Our future work will focus on this in detail, but areas of consideration include support for digital anatomy from health care advocate bodies, medical education bodies, and leading health care societies such as the Health Informatics Management Systems Society or the Australasian Institute of Digital Health. In addition, careful attention should be paid to the development of appropriate standards and quality for digital anatomy construction, design, and development. We believe that this is an exciting, emerging area that holds the promise of significant advances for both the learning and teaching of medicine as well as the practice of medicine and close by calling for more research in this key area.

## Abbreviations

AR: augmented reality
MR: mixed reality
SWOT: Strength, Weakness, Opportunity, and Threat
VR: virtual reality
